# Publisher Correction: Exploring high-quality microbial genomes by assembling short-reads with long-range connectivity

**DOI:** 10.1038/s41467-024-51890-w

**Published:** 2024-08-29

**Authors:** Zhenmiao Zhang, Jin Xiao, Hongbo Wang, Chao Yang, Yufen Huang, Zhen Yue, Yang Chen, Lijuan Han, Kejing Yin, Aiping Lyu, Xiaodong Fang, Lu Zhang

**Affiliations:** 1 Department of Computer Science, Hong Kong Baptist University, Hong Kong, China; 2 BGI Research, Shenzhen, China; 3 BGI Research, Sanya, China; 4 State Key Laboratory of Dampness Syndrome of Chinese Medicine, The Second Affiliated Hospital of Guangzhou University of Chinese, Guangzhou, China; 5 Department of Scientific Research, Kangmeihuada GeneTech Co., Ltd (KMHD), Shenzhen, China; 6 Institute for Research and Continuing Education, Hong Kong Baptist University, Shenzhen, China; 7 School of Chinese Medicine, Hong Kong Baptist University, Hong Kong, China

**Keywords:** DNA sequencing, Metagenomics

Correction to: *Nature Communications* 10.1038/s41467-024-49060-z, published online 31 May 2024

The incorrect Peer Review file was originally published with this article; it has now been replaced with the correct file. The original article has been corrected.

### Supplementary information

Peer Review file

