# Supplementary material for: Exploring high-quality microbial genomes by assembling short-reads with long-range connectivity
Source: Nat Commun. 2024 May 31;15:4631. doi: 10.1038/s41467-024-49060-z (PMC11143213; doi:10.1038/s41467-024-49060-z)
Supplement: Supplementary file 1 — Supplementary Information [file 41467_2024_49060_MOESM1_ESM.pdf]

Exploring high-quality microbial genomes by assembling short-reads with long-range connectivity

Table of Contents

Supplementary Figures 2

Supplementary Tables 8

Supplementary Notes 12

Supplementary Note 1: 10x, stLFR and TELL-Seq Sequencing 12

Supplementary Note 2: Read binning of Pangaea 15

Supplementary Note 3: Parameter search of Pangaea 17

Supplementary Note 4: Pangaea generated more NCMAGs using linked-reads than metaFlye using PacBio long-reads 18

References 19

Supplementary Figures

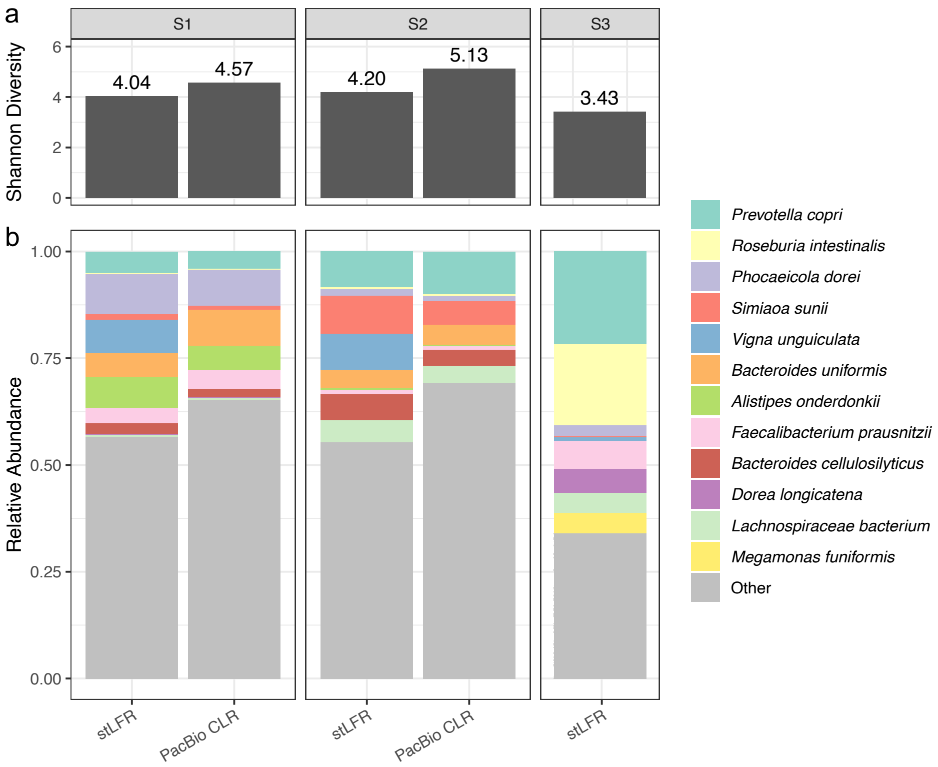

Supplementary Figure 1. The reads composition and Shannon diversity of sequencing data from different sequencing technologies on S1, S2, and S3. **a** Shannon diversity of stLFR linked-reads and PacBio CLR long-reads on S1, S2, and S3. **b** Reads composition of stLFR linked-reads and PacBio CLR long-reads on S1, S2, and S3. Source data are provided as a Source Data file.

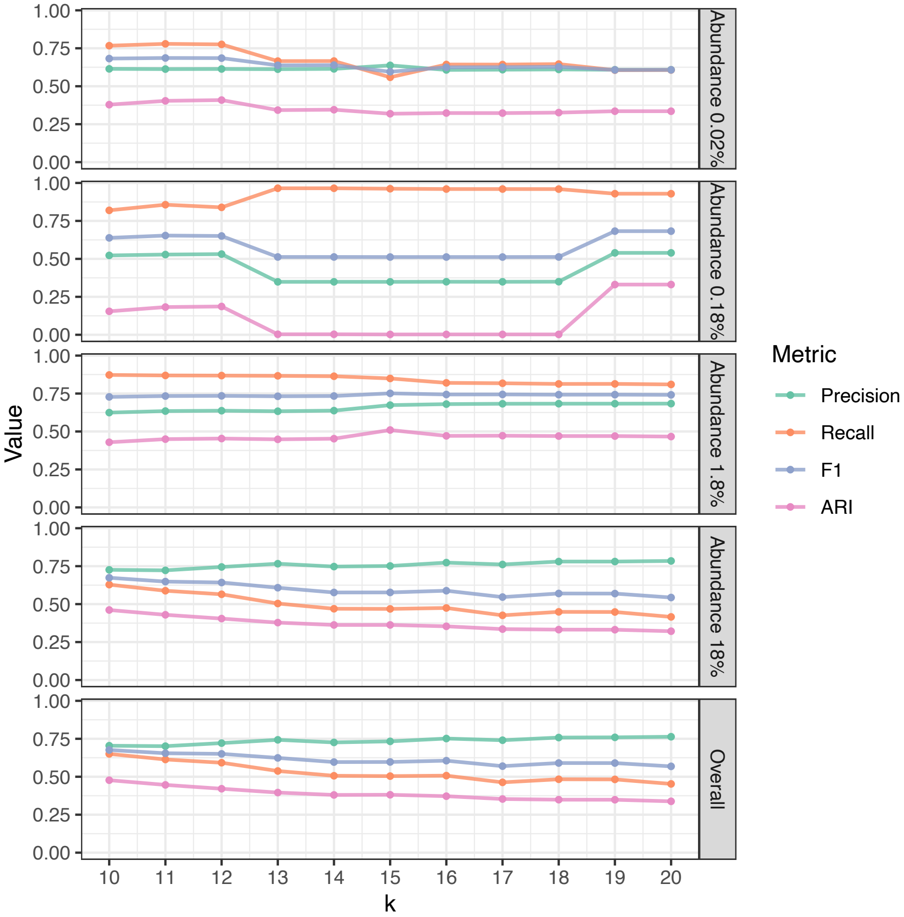

Supplementary Figure 2. The precision, recall, F1 score and adjusted rand index (ARI) values of the co-barcoded read binning using different numbers of clusters on the stLFR linked-reads of ATCC-MSA-1003. The results are organized by strains of different abundance levels. Source data are provided as a Source Data file.

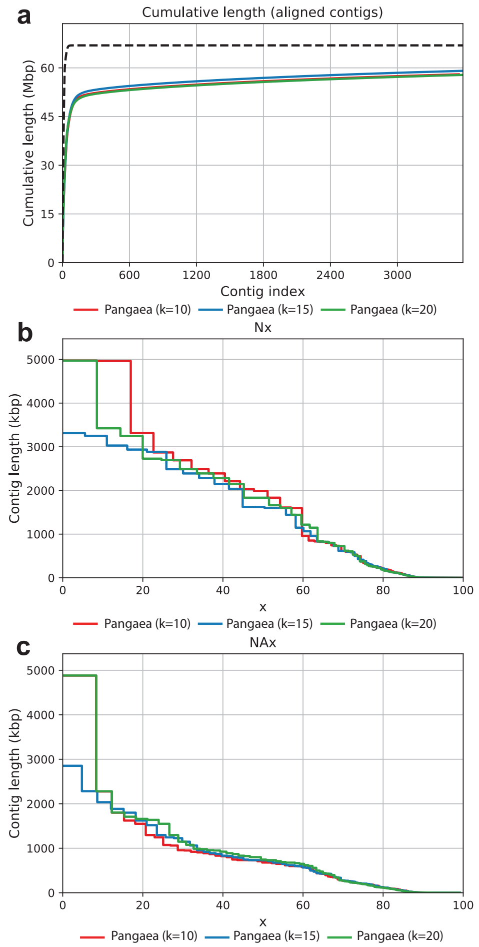

Supplementary Figure 3. The assembly statistics of Pangaea using different cluster numbers (k=10, 15, and 20) on stLFR linked-reads of the ATCC-MSA-1003 mock community. **a** Cumulative length of Pangaea assemblies using k=10, 15, and 20. **b** Nx of Pangaea assemblies using k=10, 15, and 20. **c** NAx of Pangaea assemblies using k=10, 15, and 20. Source data are provided as a Source Data file.

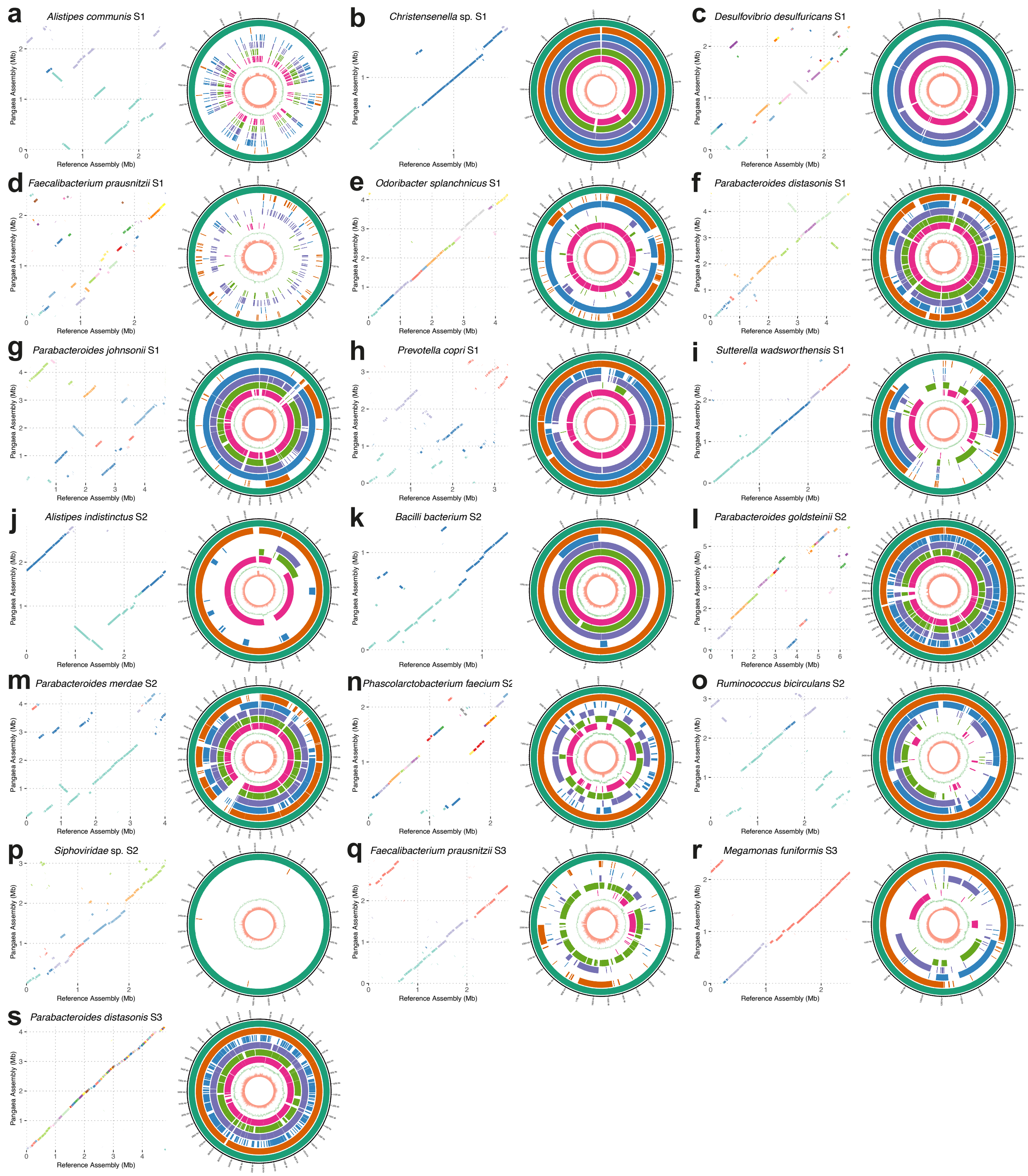

Supplementary Figure 4. Genome collinearity between the species-level NCMAGs produced by Pangaea (except for those shown in the main manuscript) and their closest reference genomes (dot plots), and comparison of different MAGs of the same species (circos plots). **a-i** The selected NCMAGs of Pangaea for S1. **j-p** The selected NCMAGs of Pangaea for S2. **q-s** The selected NCMAGs of Pangaea for S3. Pangaea obtained complete and circular NCMAGs for a, b and i. Concentric rings in the circos plots from outermost to innermost display Pangaea (dark green),

Athena (orange), Supernova (blue), cloudSPAdes (purple), MEGAHIT (light green), metaSPAdes (pink), GC-skew, and read depth of Pangaea MAGs, respectively. Long tick marks on the outer black circle indicate 100Kb intervals. For species with multiple MAGs from the same assembler, only the MAG with the highest N50 is shown. Source data are provided as a Source Data file.

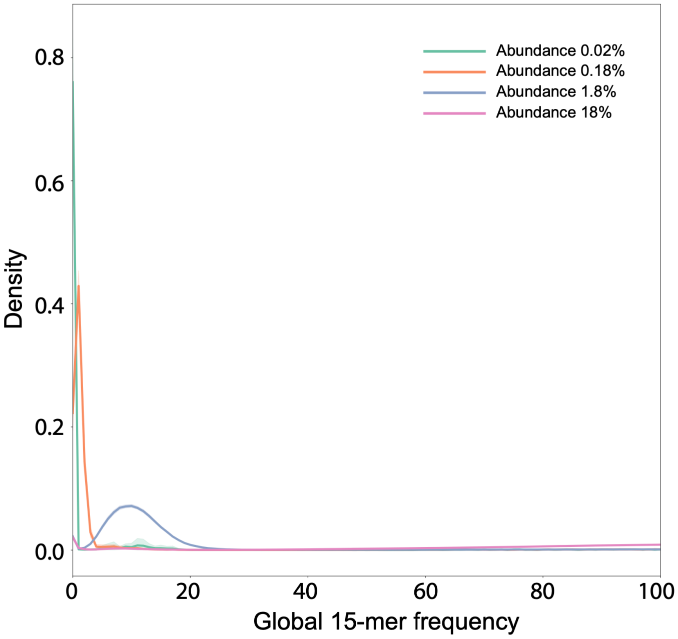

Supplementary Figure 5. The *k*-mer histograms of co-barcoded reads of different abundance levels on the stLFR linked-reads of ATCC-MSA-1003. The line for abundance 18% is truncated at the global 15-mer frequency at 100 for better visualization of the other three abundance levels.

Supplementary Tables

Supplementary Table 1. The composition and taxonomy ID of the 20 strains in the ATCC-MSA-1003 mock community.

Organism	ATCC ID	Composition	Taxonomy ID
Bacteroides vulgatus	ATCC_8482	0.02%	NCBI:txid435590
Bifidobacterium adolescentis	ATCC_15703	0.02%	NCBI:txid367928
Deinococcus radiodurans	ATCC_BAA816	0.02%	NCBI:txid243230
Enterococcus faecalis	ATCC_47077	0.02%	NCBI:txid474186
Schaalia odontolytica	ATCC_17982	0.02%	NCBI:txid411466
Acinetobacter baumannii	ATCC_17978	0.18%	NCBI:txid400667
Cutibacterium acnes	ATCC_11828	0.18%	NCBI:txid1091045
Helicobacter pylori	ATCC_700392	0.18%	NCBI:txid85962
Lactobacillus gasseri	ATCC_33323	0.18%	NCBI:txid324831
Neisseria meningitidis	ATCC_BAA335	0.18%	NCBI:txid122586
Bacillus cereus	ATCC_10987	1.8%	NCBI:txid222523
Clostridium beijerinckii	ATCC_35702	1.8%	NCBI:txid864803
Pseudomonas aeruginosa	ATCC_9027	1.8%	NCBI:txid287
Staphylococcus aureus	ATCC_BAA1556	1.8%	NCBI:txid451515
Streptococcus agalactiae	ATCC_BAA611	1.8%	NCBI:txid208435
Escherichia coli	ATCC_700926	18%	NCBI:txid511145
Porphyromonas gingivalis	ATCC_33277	18%	NCBI:txid431947
Rhodobacter sphaeroides	ATCC_17029	18%	NCBI:txid349101
Staphylococcus epidermidis	ATCC_12228	18%	NCBI:txid176280
Streptococcus mutans	ATCC_700610	18%	NCBI:txid210007

Supplementary Table 2. The sequencing statistics of all the datasets used in the manuscript.

Microbial community	Sequencing technology	Read length	Total sequencing size (Gb)	Shannon diversity
ZYMO	stLFR	100	143.04	0.50
ZYMO	ONT	4,504 (average)	2.48 (downsampled)	Not needed
ATCC-MSA-1003	10x	150	100.38	1.83
ATCC-MSA-1003	TELL-Seq	146	173.28	1.89
ATCC-MSA-1003	stLFR	100	132.95	1.91
ATCC-MSA-1003	PacBio CLR	8,875 (average)	1.27 (downsampled)	Not needed
CAMI-high	stLFR	100	100.01	4.57
CAMI-high	ONT	4,122 (average)	7.38	Not needed
Human gut microbiome (S1)	stLFR	100	136.60	4.08
Human gut microbiome (S1)	PacBio CLR	8,878 (average)	6.26	Not needed
Human gut microbiome (S2)	stLFR	100	131.59	3.77
Human gut microbiome (S2)	PacBio CLR	8,973 (average)	8.39	Not needed
Human gut microbiome (S3)	stLFR	100	50.74	3.51

Supplementary Table 3. Assembly statistics for different linked-read sequencing technologies on the ATCC-MSA-1003 mock community.

	Pangaea TELL-Seq	Pangaea stLFR	Pangaea 10x
Total assembly length	61,990,266	59,485,233	58,860,253
Genome fraction (%)	82.63	84.43	83.23
Longest alignment	4,968,123	2,853,278	58,622,163
Overall N50	1,360,322	1,619,916	1,033,793
Overall NA50	649,672	731,990	483,416
NGA50 per strain	887,107	677,353	441,029
NA50 per strain	838,457	628,059	421,157

Supplementary Table 4. The composition of the 10 strains in the ZYMO mock community.

Organism	Composition
Listeria monocytogenes	89.1%
Pseudomonas aeruginosa	8.9%
Bacillus subtilis	0.89%
Saccharomyces cerevisiae	0.89%
Escherichia coli	0.089%
Salmonella enterica	0.089%
Lactobacillus fermentum	0.0089%
Enterococcus faecalis	0.00089%
Cryptococcus neoformans	0.00089%
Staphylococcus aureus	0.000089%

Supplementary Notes

Supplementary Note 1: 10x, stLFR and TELL-Seq Sequencing

We compared the characteristics of 10x, stLFR and TELL-Seq linked-reads on the mock community. 10x linked-reads generated the lowest number of unique barcodes (2.31 million; Supplementary Table 5), where the values of stLFR and TELL-Seq were 45.38 and 16.61 million, respectively (Supplementary Table 5). We reconstructed the physical long fragments and calculated the number of fragments per barcode ($N_{F/B}$) for the three technologies by mapping the linked-reads to reference genomes. 10x linked-reads had the lowest barcode specificity with $N_{F/B}$ of 16.61 (Supplementary Table 5), where the values of stLFR and TELL-Seq linked-reads were much lower, which were 1.54 and 4.26, respectively (Supplementary Table 5). We aggregated the barcodes based on long fragments they involved and found highest fraction is 6.41% for 14 long fragments. The highest fractions for both stLFR (62.98%) and TELL-Seq (27.05%) linked-reads were matching to one long fragment (Supplementary Fig. 6).

10x and stLFR linked-reads generated comparable lengths of the long fragments, that were longer than those from TELL-Seq linked-reads (Supplementary Fig. 7); the weighted average of long fragment lengths ($W_{\mu_{FL}}$) for 10x, stLFR and TELL-Seq linked-reads were 17.02Kb, 15.68Kb and 11.85Kb, respectively (Supplementary Table 5). All the three linked-read sequencing technologies had shallow depth of short-reads for long fragments, with similar distributions (Supplementary Fig. 8) and comparable average value (C_R: 10x = 0.21, stLFR = 0.20, TELL-Seq = 0.17; Supplementary Table 5).

Further, we investigated the insert sizes of linked-reads generated from the three sequencing technologies. stLFR (246 bp) and TELL-Seq (204 bp) linked-reads obtained lower median insert sizes than 10x linked-reads (339 bp). We observed the histograms of the insert sizes from stLFR and TELL-Seq linked-reads were more biased than that from 10x linked-reads, and the curve of histogram from TELL-Seq linked-reads had frequent vibrations (Supplementary Fig. 9).

Supplementary Table 5. The statistics of 10x, TELL-Seq and stLFR linked-reads.

	10x linked-reads	stLFR linked-reads	TELL-Seq linked-reads
Number of barcodes	2,305,295	45,381,261	16,609,060
Number of fragments per barcode ($N_{F/B}$)	16.61	1.54	4.26
Weighted fragment length ($W_{\mu_{FL}}$, bp)	17,022	15,683	11,848

Read depth per fragment (C_R)	0.21	0.20	0.17
Median insert size (bp)	339	246	204

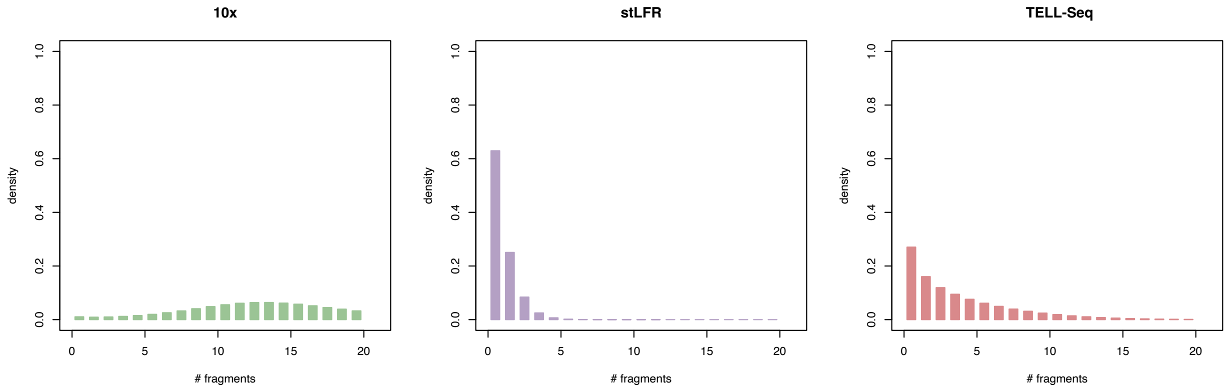

Supplementary Figure 6. The distributions of the number of physical long fragments per barcode.

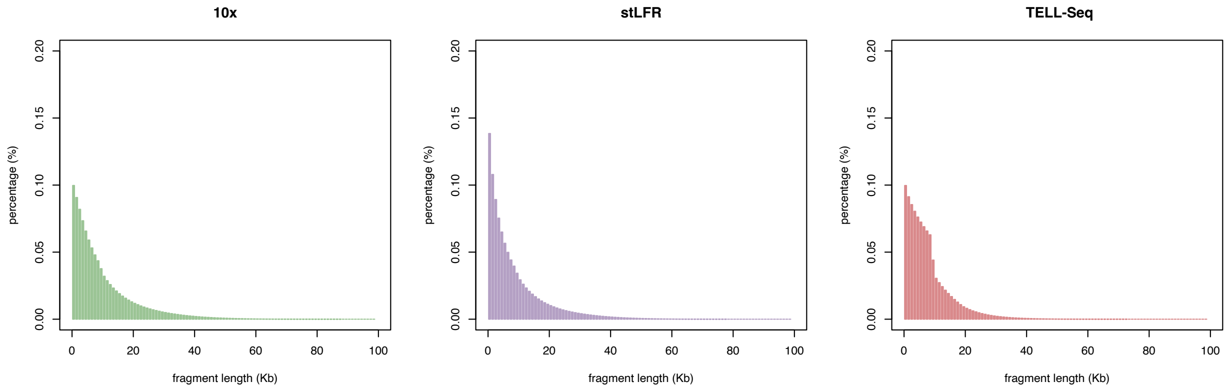

Supplementary Figure 7. The distributions of the lengths of physical long fragments.

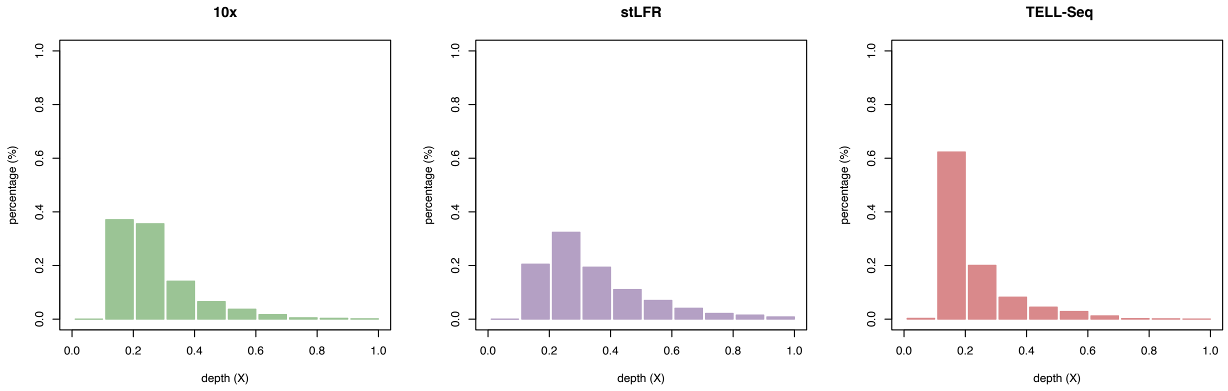

Supplementary Figure 8. The distributions of read depths per long fragments.

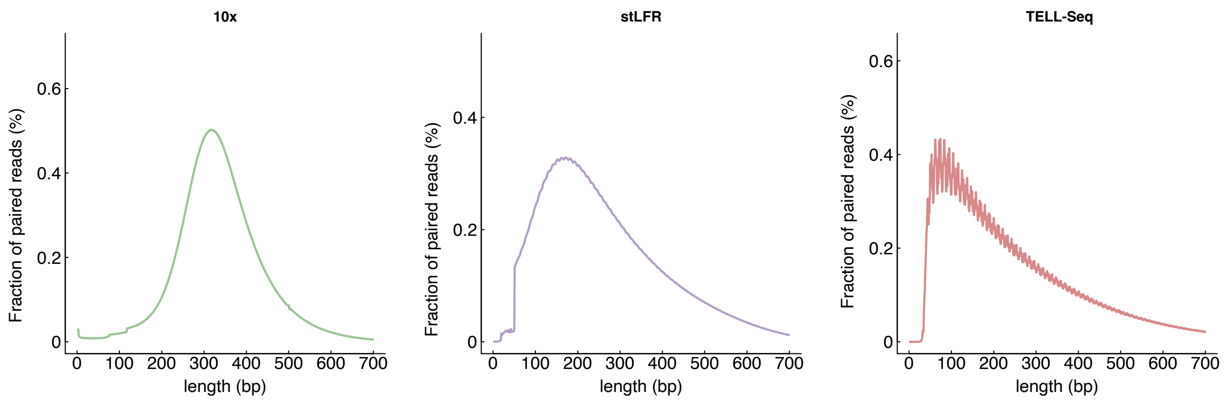

Supplementary Figure 9. The distributions of insert sizes for 10x, stLFR and TELL-Seq linked-reads.

Supplementary Note 2: Read binning of Pangaea

1. Architecture of VAE

We used VAE to learn the low-dimensional representation of the co-barcoded linked-reads based on their abundances ($\boldsymbol{X_A}$) and TNF ($\boldsymbol{X_T}$) features (Supplementary Fig. 10). The input of VAE was the concatenation of L1-normalized $\boldsymbol{X_A}$ (400 dimensions) and $\boldsymbol{X_T}$ (136 dimensions). In the encoder, the input features were transformed by two fully connected hidden layers with 512 neurons for each, and compressed into two 32-dimensional latent parameters μ and σ for Gaussian distribution. We sampled the latent embedding $\boldsymbol{Z}$ from the Gaussian distribution $N(\mu, \sigma^2)$. To make the sampling process trainable for μ and σ, we used the reparameterization trick, that calculated $\boldsymbol{Z}$ by $\boldsymbol{Z} = \mu + \sigma \circ \epsilon, \epsilon \sim N(0,1)$. In the decoder, we used two fully connected hidden layers with the same settings as the encoder, and reconstructed the input features using an output layer with 516 neurons. The output vector was split to the reconstructed abundances ($\boldsymbol{\hat{X}_A}$) and TNF ($\boldsymbol{\hat{X}_T}$) features according to their corresponding dimensions. Both $\boldsymbol{\hat{X}_A}$ and $\boldsymbol{\hat{X}_T}$ were transformed by softmax before output. To increase the stability of training, each hidden layer in the encoder and decoder was followed by a batch normalization layer and a dropout layer with P=0.2. Our batch size and learning rate were set to 2,048 and 0.005. We exploited weight decay of 1e-4 by default to avoid overfitting and used the early stopping that stopped the training when the loss started to increase, which substantially reduced the training time.

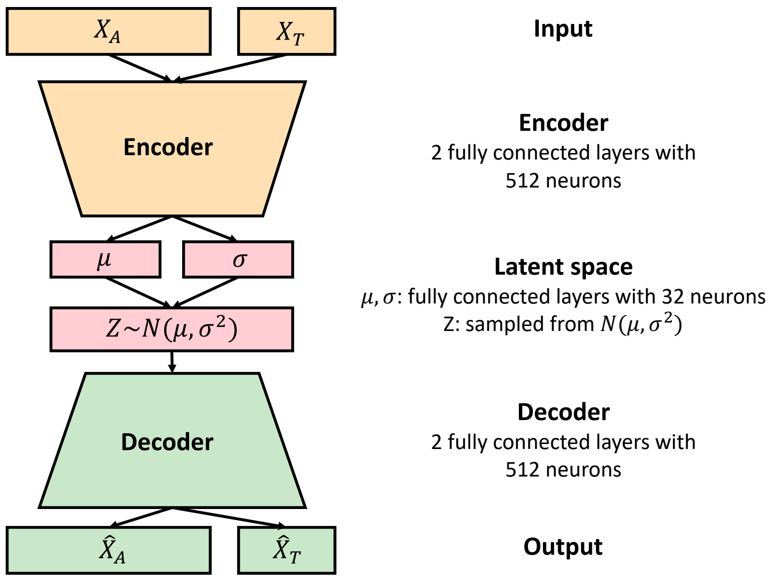

Supplementary Figure 10. The architecture of VAE used in Pangaea.

2. Comparing read binning of Pangaea with other read binning tools

As there were no available binning tools for linked-reads, we compared the VAE-based binning algorithm in Pangaea with two existing long-read binning tools, METABCC-LR (v2.0.0)[1]

and LRBinner (v2.1)[2] on ATCC-MSA-1003. In order to enable these long-read binning tools on linked-reads, the co-barcoded reads were connected using a single "N" into long-reads. As the long-read binners will skip k-mers containing N (see code in METABCC-LR; the same for LRBinner), this process will not impact the k-mer frequency of the co-barcoded reads for long-read binning tools. LRBinner failed to finish the binning task within three weeks with 100 threads, so we excluded it for further comparison. The VAE-based binning algorithm achieved a higher overall F1 score and adjusted rand index (ARI) than METABCC-LR (Pangaea: F1 = 0.6144, ARI = 0.3764; METABCC-LR: F1 = 0.5887; ARI = 0.1704; Supplementary Table 6). The VAE-based binning particularly improved the binning performance for low-abundance microbes (abundance: 0.02%), achieving an F1 score of 0.6073 and ARI of 0.4624. The performance of METABCC-LR was almost the same as that of random binning (Supplementary Table 6).

Supplementary Table 6. The F1 scores and ARIs of the binning algorithm of Pangaea and METABCC-LR on the stLFR linked-reads of ATCC-MSA-1003.

	Binning of Pangaea	METABCC-LR
Number of clusters	15	2
F1 – abundance 0.02%	0.6073	0.4624
ARI – abundance 0.02%	0.3246	0
F1 – abundance 0.18%	0.5125	0.5167
ARI – abundance 0.18%	0.0022	0.0140
F1 – abundance 1.8%	0.7500	0.6155
ARI – abundance 1.8%	0.5069	0.1740
F1 – abundance 18%	0.5960	0.6351
ARI – abundance 18%	0.3581	0.2019
F1 - overall	0.6144	0.5887
ARI - overall	0.3764	0.1704

Supplementary Note 3: Parameter search of Pangaea

1. α and β for the weights in the loss function

We searched a range of hyper-parameters α and β on the stLFR linked-reads dataset from ATCC-MSA-1003. The overall adjusted rand index (ARI) and F1 did not vary much when changing β, so we used β =0.015 because the opposite trends in overall precision and ARI were observed after β =0.015 (Supplementary Fig. 11a). The lowest α resulted in the highest overall precision, recall, F1, and ARI (Supplementary Fig. 11b). Therefore, we adopted a low value of α =0.1. The w_T is much larger than w_A, which means TNF is a more important feature than the *k*-mer histogram in read binning. This might be because the *k*-mer-histograms using a larger *k* (*k*=15) than TNF (*k*=4) are more likely to be influenced by sequencing errors and contain more noise. We used a small w_{KL}, which was consistent with the previous study that applies VAE in contig binning tasks[3].

2. Thresholding T for multi-thresholding reassembly

We investigated the impact of the range of thresholds T on the final assembly results using the TELL-Seq dataset of ATCC-MSA-1003 (Supplementary Data 4). We compared the assembly results with and without the multi-thresholding reassembly. We observed T = {10, 30} obtained the highest N50 (1.36MB), the highest total length (61.99Mb), and the highest total length of long contigs > 10Kb (55.35Mb). Involving higher thresholds did not bring much improvement and would need a longer time to run (Supplementary Data 4).

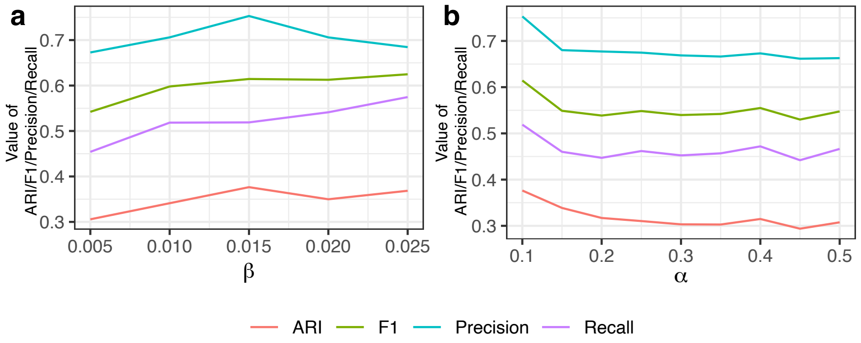

Supplementary Figure 11. The binning performance on the stLFR linked-reads dataset of ATCC-MSA-1003 using different parameters in the loss function of VAE. **a** Binning performance using different α (β is fixed at 0.015). **b** Binning performance using different β (α is fixed at 0.1).

Supplementary Note 4: Pangaea generated more NCMAGs using linked-reads than metaFlye using PacBio long-reads

We compared contigs from Pangaea using linked-reads with the contigs produced by metaFlye using PacBio CLR long-reads on S1 and S2 (Supplementary Table 2; Supplementary Fig. 4). Although metaFlye generated contigs with higher N50s, Pangaea produced a substantially greater total assembly length for both S1 (Pangaea = 488.19Mb, metaFlye = 243.88Mb) and S2 (Pangaea = 408.82Mb, metaFlye = 256.78Mb; Supplementary Table 7).

Then we binned the assemblies using MetaBAT2 and counted the number of NCMAGs from the binning results. Pangaea generated significantly more NCMAGs than metaFlye (Pangaea = 41, metaFlye = 16; Supplementary Fig. 12a), especially those with N50s smaller than 1Mb (Pangaea = 29, metaFlye = 4; Supplementary Fig. 12b,d) and read depths lower than 300x (Pangaea = 27, metaFlye = 0; Supplementary Fig. 12c), whereas Pangaea and metaFlye obtained comparable numbers of NCMAGs with N50 larger than 1Mb (Pangaea = 12, metaFlye = 12; Supplementary Fig. 12b). Pangaea also assembled more ORF clusters (Pangaea = 522.81K, metaFlye = 169.79K), 5s rRNAs (Pangaea = 612, metaFlye = 400), 16s rRNAs (Pangaea = 690, metaFlye = 492), 23s rRNAs (Pangaea = 700, metaFlye = 491), plasmid contigs (Pangaea = 3,376, metaFlye = 218) and viral contigs (Pangaea = 1,707, metaFlye = 61) than metaFlye (Supplementary Table 8).

Supplementary Table 7. Assembly statistics for Pangaea and metaFlye on S1 and S2.

	Human gut microbiome (S1)		Human gut microbiome (S2)	
	Pangaea	metaFlye	Pangaea	metaFlye
Total assembly length	488,785,611	243,883,392	408,819,148	256,776,331
Largest contig	2,394,379	3,388,254	2,877,256	4,327,156
N50	64,394	168,442	188,161	239,008

Supplementary Table 8. The total number of ORF clusters, rRNAs, plasmid contigs (>1Kb), and viral contigs (>1Kb) of Pangaea and metaFlye on S1 and S2.

	Pangaea	metaFlye
ORF clusters	522,812	169,785
5S rRNA	612	440
16s rRNA	690	492
23s rRNA	700	491
Plasmid contigs (>1Kb)	3,376	218

Viral contigs (>1Kb)	1,707	61

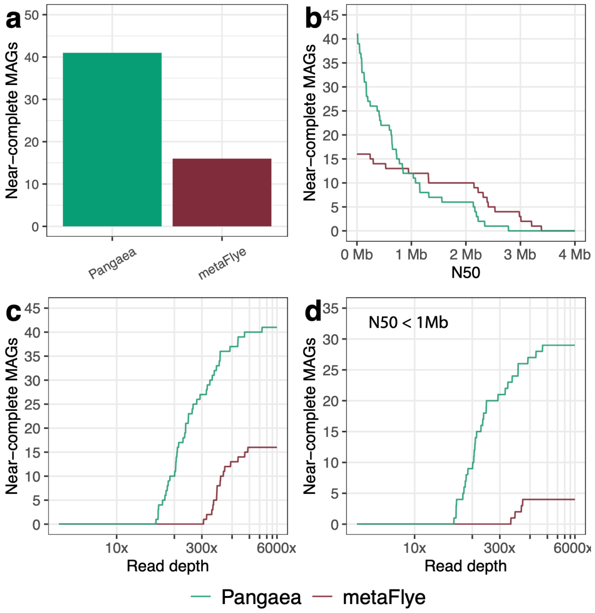

Supplementary Figure 12. The number of NCMAGs of Pangaea and metaFlye on S1 and S2. **a** the number of NCMAGs of Pangaea and metaFlye on S1 and S2. **b** the number of NCMAGs of Pangaea and metaFlye on S1 and S2 with a minimum value of N50. **c** the number of NCMAGs of Pangaea and metaFlye on S1 and S2 with a maximum value of read depth. **d** the number of NCMAGs (N50 < 1Mb) of Pangaea and metaFlye on S1 and S2 with a maximum value of read depth.

References

1 Wickramarachchi, A., Mallawaarachchi, V., Rajan, V. & Lin, Y. Metabcc-lr: meta genomics b inning by c overage and c omposition for l ong r eads. *Bioinformatics* **36**, i3-i11 (2020).

2 Wickramarachchi, A. & Lin, Y. in *21st International Workshop on Algorithms in Bioinformatics (WABI 2021).* (Schloss Dagstuhl-Leibniz-Zentrum für Informatik).

3 Nissen, J. N. *et al.* Improved metagenome binning and assembly using deep variational autoencoders. *Nat Biotechnol* **39**, 555-560 (2021). https://doi.org:10.1038/s41587-020-00777-4
