# Supplementary material for: Exploring high-quality microbial genomes by assembling short-reads with long-range connectivity
Source: Nat Commun. 2024 May 31;15:4631. doi: 10.1038/s41467-024-49060-z (PMC11143213; doi:10.1038/s41467-024-49060-z)
Supplement: Supplementary file 3 — Description of Additional Supplementary Files [file 41467_2024_49060_MOESM3_ESM.pdf]

File name: Supplementary Data 1
Description: The impact of VAE-based binning algorithm adopted by Pangaea on the final assembly results using linked-reads of ATCC-MSA-1003, CAMI-high, S1, S2, and S3.

File name: Supplementary Data 2
Description: The completeness, contamination, number of tRNAs, number of 5s rRNAs, 16s rRNAs, and 23s rRNAs of the MAGs produced from Pangaea assemblies with co-barcoded read binning and without co-barcoded read binning, on CAMI-high, S1, S2, and S3.

File name: Supplementary Data 3
Description: The assembly statistics of Pangaea using different parameters of k on CAMI-High.

File name: Supplementary Data 4
Description: The impact of different ranges of abundance thresholds (Ts) on the final assembly using the TELL-Seq linked-reads of the ATCC-MSA-1003 mock community.

File name: Supplementary Data 5
Description: The genome fractions for all genomes and circular elements of CAMI-high from the Pangaea assemblies with multi-thresholding reassembly and without multi-thresholding reassembly.

File name: Supplementary Data 6
Description: The per-strain NA50 and NGA50 generated by different assembly tools for the 15 strains with abundances higher than 0.1% using TELL-Seq, stLFR, and 10x Genomics linked-reads (or corresponding barcode-removed short-reads) from ATCC-MSA-1003.

File name: Supplementary Data 7
Description: The genome fractions generated by different assembly tools for the five strains with abundances of 0.02% using TELL-Seq, stLFR, and 10x Genomics linked-reads (or corresponding barcode-removed short-reads) from ATCC-MSA-1003.

File name: Supplementary Data 8
Description: The genome fractions, per-strain NA50 and NGA50 generated by different assembly tools for the 10 strains using the stLFR linked-reads (or corresponding barcode-removed short-reads) from ZYMO.

File name: Supplementary Data 9
Description: The statistics of MAGs generated for the three human gut microbiomes by all assemblers.

File name: Supplementary Data 10
Description: The closest reference genomes of the NCMAGs with species-level annotations generated by all assemblers and their alignment statistics reported by GTDB-Tk (v2.1.0).

File name: Supplementary Data 11
Description: The assembly statistics of all assemblers on the subsampled linked-read datasets (5Gb, 10Gb, 20Gb, 50Gb, and 100Gb) or corresponding barcode-removed short-reads of ATCC-MSA-1003, CAMI-high, and S1.

File name: Supplementary Data 12

Description: The genome fractions, total assembly length for contigs longer than 10Kb, per-strain NA50 and NGA50 by different assemblers using short-reads, short-reads with virtual barcodes, or short- and long-reads from ATCC-MSA-1003 and ZYMO mock communities.

File name: Supplementary Data 13
Description: The statistics of the number of misassemblies per 1Mb, the number of chimeric assemblies per 1Mb, and sequence identities of all assemblers on ATCC-MSA-1003, ZYMO, and CAMI-high mock communities.
