# Supplementary material for: Exploring high-quality microbial genomes by assembling short-reads with long-range connectivity
Source: Nat Commun. 2024 May 31;15:4631. doi: 10.1038/s41467-024-49060-z (PMC11143213; doi:10.1038/s41467-024-49060-z)
Supplement: Supplementary file 17 — Reporting Summary [file 41467_2024_49060_MOESM17_ESM.pdf]

nature portfolio

Corresponding author(s): Lu Zhang

Last updated by author(s): May 9, 2024

Reporting Summary

Nature Portfolio wishes to improve the reproducibility of the work that we publish. This form provides structure for consistency and transparency in reporting. For further information on Nature Portfolio policies, see our Editorial Policies and the Editorial Policy Checklist.

Statistics

For all statistical analyses, confirm that the following items are present in the figure legend, table legend, main text, or Methods section.

n/a	Confirmed	
☐	☒	The exact sample size (*n*) for each experimental group/condition, given as a discrete number and unit of measurement
☐	☒	A statement on whether measurements were taken from distinct samples or whether the same sample was measured repeatedly
☐	☒	The statistical test(s) used AND whether they are one- or two-sided<br>*Only common tests should be described solely by name; describe more complex techniques in the Methods section.*
☒	☐	A description of all covariates tested
☒	☐	A description of any assumptions or corrections, such as tests of normality and adjustment for multiple comparisons
☐	☒	A full description of the statistical parameters including central tendency (e.g. means) or other basic estimates (e.g. regression coefficient) AND variation (e.g. standard deviation) or associated estimates of uncertainty (e.g. confidence intervals)
☐	☒	For null hypothesis testing, the test statistic (e.g. *F*, *t*, *r*) with confidence intervals, effect sizes, degrees of freedom and *P* value noted<br>*Give P values as exact values whenever suitable.*
☒	☐	For Bayesian analysis, information on the choice of priors and Markov chain Monte Carlo settings
☒	☐	For hierarchical and complex designs, identification of the appropriate level for tests and full reporting of outcomes
☒	☐	Estimates of effect sizes (e.g. Cohen's *d*, Pearson's *r*), indicating how they were calculated

Our web collection on statistics for biologists contains articles on many of the points above.

Software and code

Policy information about availability of computer code

Data collection: No software was used for data collection.

Data analysis:

Reads preprocessing:
LRTK (v1.7)
Long Ranger (v2.2.0)
stLFR read demux (Git version 3ecaa6b)

Assembly:
MEGAHIT (v1.2.9)
metaSPAdes (v3.15.3)
cloudSPAdes (v3.12.0-dev)
Athena (v1.3)
Supernova (v2.1.1)
metaFlye (v2.8)
hybridSPAdes (v3.15.3)
OPERA-MS (v0.8.3)
quickmerge (v0.3)

Contig binning and evaluation:

MetaQUAST (v5.0.2)
R package stats (v4.4.0)
BWA-MEM (v0.7.17)
SAMtools (v1.9)
MetaBat2 (v2.12.1)
VAMB (v3.0.3)
CheckM (v1.1.2)
ARAGORN (v1.2.38)
barrnap (v0.9)
Kraken2 (v2.1.2)

Our custom code developed in the study: https://github.com/ericcombiolab/Pangaea

For manuscripts utilizing custom algorithms or software that are central to the research but not yet described in published literature, software must be made available to editors and reviewers. We strongly encourage code deposition in a community repository (e.g. GitHub). See the Nature Portfolio guidelines for submitting code & software for further information.

Data

Policy information about availability of data

All manuscripts must include a data availability statement. This statement should provide the following information, where applicable:

- Accession codes, unique identifiers, or web links for publicly available datasets
- A description of any restrictions on data availability
- For clinical datasets or third party data, please ensure that the statement adheres to our policy

The 10x Genomics linked-reads of the ATCC-MSA-1003 mock community used in this study are available in the NCBI SRA database under accession code SRR12283286 [https://www.ncbi.nlm.nih.gov/sra/SRR12283286]. The stLFR and TELL-Seq sequencing data of ATCC-MSA-1003 generated in this study have been deposited in the NCBI SRA database under accession code SRR21422848 [https://www.ncbi.nlm.nih.gov/sra/SRR21422848] and SRR21422847 [https://www.ncbi.nlm.nih.gov/sra/SRR21422847], respectively. The stLFR sequencing data of the three human gut microbiomes (S1, S2, and S3) generated in this study have been deposited in the NCBI SRA database under accession code SRR28959570 [https://www.ncbi.nlm.nih.gov/sra/SRR28959570], SRR28959569 [https://www.ncbi.nlm.nih.gov/sra/SRR28959569], and SRR28959571 [https://www.ncbi.nlm.nih.gov/sra/SRR28959571], respectively. The MAGs generated by Pangaea from S1, S2, and S3 in this study have been deposited in the European Nucleotide Archive (ENA) project under accession code PRJEB65432 [https://www.ebi.ac.uk/ena/browser/view/PRJEB65432]. The PacBio CLR long-reads of ATCC-MSA-1003, S1 and S2 used in this study are available in the NCBI SRA database under accession code SRR12371719 [https://www.ncbi.nlm.nih.gov/sra/SRR12371719], SRR19505636 [https://www.ncbi.nlm.nih.gov/sra/SRR19505636], and SRR19505632 [https://www.ncbi.nlm.nih.gov/sra/SRR19505632], respectively. The ONT long-reads of ZYMO used in this study are available in the NCBI SRA database under accession code ERR3152366 [https://www.ncbi.nlm.nih.gov/sra/ERR3152366]. Source data are provided with this paper.

Research involving human participants, their data, or biological material

Policy information about studies with human participants or human data. See also policy information about sex, gender (identity/presentation), and sexual orientation and race, ethnicity and racism.

Reporting on sex and gender	All three individuals are female. The samples of the three individuals (S1, S2, and S3) were collected at the ages of 36, 32, and 30, respectively. Sex or gender was not considered in the study design. Sex or gender of participants was determined based on self-report. No sex- and gender-based analyses was performed, this was because the relation between human gut microbiomes and the gender was beyond the scope of this study.
Reporting on race, ethnicity, or other socially relevant groupings	All three individuals are Chinese.
Population characteristics	The individuals involved in this study are healthy individuals.
Recruitment	The individuals were randomly selected from the staffs in Kangmeihuada GeneTech Co., Ltd (KMHD). Participant compensation was not applied.
Ethics oversight	The study complies with all relevant ethical regulations and was approved by the Ethics Committee of BGI (BGI-IRB 20145). We have received written informed consent from the human participants in this study.

Note that full information on the approval of the study protocol must also be provided in the manuscript.

Field-specific reporting

Please select the one below that is the best fit for your research. If you are not sure, read the appropriate sections before making your selection.

[x] Life sciences [ ] Behavioural & social sciences [ ] Ecological, evolutionary & environmental sciences

For a reference copy of the document with all sections, see nature.com/documents/nr-reporting-summary-flat.pdf

Life sciences study design

All studies must disclose on these points even when the disclosure is negative.

Sample size	Sample size for real microbiomes was 3. No sample size calculation was performed. Sample size was determined based on referencing the previous study (https://doi.org/10.1038/nbt.4266), where two human participants were enrolled. Three real microbial communities were sufficient because we also conducted thorough testing on mock communities, including ATCC-MSA-1003, ZYMO, and CAMI-high. We are confident that our approach can be applied to metagenomic data from different sources. All attempts at all the datasets were successful.
Data exclusions	No data was excluded.
Replication	One library was sequenced from each sample
Randomization	For all experiments, the participants were randomly chosen.
Blinding	The Investigators were not blinded to allocation during all experiments and all outcome assessment. No different treatments were given to different participants during experiments and outcome assessment. Therefore, blinding was not relevant to our study.

Reporting for specific materials, systems and methods

We require information from authors about some types of materials, experimental systems and methods used in many studies. Here, indicate whether each material, system or method listed is relevant to your study. If you are not sure if a list item applies to your research, read the appropriate section before selecting a response.

Materials & experimental systems

n/a	Involved in the study	
☒	☐	Antibodies
☒	☐	Eukaryotic cell lines
☒	☐	Palaeontology and archaeology
☒	☐	Animals and other organisms
☒	☐	Clinical data
☒	☐	Dual use research of concern
☒	☐	Plants

Methods

n/a	Involved in the study	
☒	☐	ChIP-seq
☒	☐	Flow cytometry
☒	☐	MRI-based neuroimaging
